# Factors Influencing Utilization of Preventive Health Services in Primary Health Care in the Republic of Serbia

**DOI:** 10.3390/ijerph18063042

**Published:** 2021-03-16

**Authors:** Slavka Mitričević, Janko Janković, Željka Stamenković, Vesna Bjegović-Mikanović, Marko Savić, Dejana Stanisavljević, Stefan Mandić-Rajčević

**Affiliations:** 1 Cabinet of Minister without Portfolio in Charge of Demography and Population Policy, 11000 Belgrade, Serbia; slavka.mitricevic@mdpp.gov.rs; 2 Institute of Social Medicine, Faculty of Medicine, University of Belgrade, 11000 Belgrade, Serbia; janko.jankovic@med.bg.ac.rs (J.J.); zeljka.stamenkovic@med.bg.ac.rs (Ž.S.); vesna.bjegovic-mikanovic@med.bg.ac.rs (V.B.-M.); 3 Centre-School of Public Health and Health Management, Faculty of Medicine, University of Belgrade, 11000 Belgrade, Serbia; 4 Department for Medical Statistics and Informatics, Faculty of Medicine, University of Belgrade, 11000 Belgrade, Serbia; marko.savic@med.bg.ac.rs (M.S.); dejana.stanisavljevic@med.bg.ac.rs (D.S.)

**Keywords:** factors, preventive health services, primary health care, utilization

## Abstract

The use of preventive health services is a long-term health investment due to its potential to help individuals to take care of their health. This study aimed to explore the availability and performance of health services in primary health care (PHC) in the domain of general practice (GP), pediatrics, and gynecology, as well as to analyze the influence of sociodemographic and health determinants on the utilization of preventive health services. This descriptive study used data from the National Health Insurance Fund and the Statistical Office of the Republic of Serbia for 2015 and included 149 independent PHC units. The relationship between the utilization of preventive services and sociodemographic and health characteristics of the population was analyzed by bivariate and multivariate linear regression models. The higher health expenditure per capita and noncommunicable diseases mortality rate were, the more preventive health services were provided by a chosen GP. Children with a higher completion rate of primary school (*p* = 0.024), higher health expenditure (*p* = 0.017), and higher life expectancy at birth (*p* = 0.041) had more preventive health services. The fertility rate was positively associated with the number of preventive health services per 1000 women (*p* = 0.033). Our findings should serve as a starting point for where efforts should be made to achieve better health outcomes.

## 1. Introduction

Contemporary global policies acknowledge the holistic approach to health through considering social determinants of health, along with the organization, functioning, and financing of health care. This approach points to the responsibility of the whole society, which is reflected in the syntagma “health in all policies” [1,2]. The United Nations Agenda for Sustainable Development 2030 is one of the global policies that has emphasized health as a key component for development, especially in its third Sustainable Development Goal that explicitly focuses on health and access to quality health care through adequately provided preventive health services [3,4].

The real value of prevention is to ensure that a disease does not occur. In addition to preventive services provided at the primary level of health care (check-ups, patient counseling, and screening tests), there are measures and activities conducted at the local or community level and connected to the wider health determinants that are the responsibility not only of doctors and the health care system but also of people and authorities from different sectors in a society [5]. While care services may be able to alter the trajectory of a condition, a patient’s natural history and issues prior to accessing care are related to the initiation of the disease process.

Low- and middle-income countries, including the Republic of Serbia, have faced an increase in chronic non-communicable diseases (NCDs) due to increasing exposure to various risk factors [6,7]. In the Republic of Serbia, cardiovascular diseases account for 54% of all deaths, while almost every fourth person dies from malignant diseases (22%) [8]. The provision of preventive services may seem as an additional financial burden on the existing system [9]. However, as World Health Organization stated, preventing chronic diseases is a vital investment in health, and different approaches to manage chronic diseases may give rise to economic benefits [10]. For example, it is estimated that if the risk factors were eliminated, over 80% of cardiovascular diseases and diabetes mellitus type 2 and more than 40% of malignant diseases could be prevented, and the implementation of preventive measures has proven to be cost-efficient in all counties of the world [10]. Preventive services benefit both the population’s health and healthcare systems through reduced treatment costs. The use of preventive health services is recognized as a long-term health investment due to its potential to help individuals to take care of their health and wellbeing and to diminish chances of getting a disease, especially NCDs [11,12].

Health care in Republic of Serbia is based on a system of mandatory social health insurance financed by salary contributions paid by employers and employees to the Republic Health Insurance Fund, which also guarantees health insurance coverage to unemployed, internally displaced people, refugees, and marginalized population groups. Medical services such as specialist treatment, hospitalization, prescriptions, services during pregnancy and childbirth, and rehabilitation are covered by a state fund [9]. Though the Republic of Serbia spends 8.8% of its GDP on health in 2017, which is one of the highest percentages in the Balkan region, the expenditure directed to preventive health care remains low. Preventive services account for around 7.5% of total health expenditure, and the accent is still on the curative rather than preventive services [9]. In the Republic of Serbia, preventive services are almost exclusively delivered through primary health care (PHC) that is equally geographically distributed by the territorial level of municipalities and represent the first level of contact of an individual, family, and community with the healthcare system. PHC is characterized by chosen physicians or “gatekeepers” in the healthcare system who, together with nurses, form a team that provides preventive services to insured patients in the area of general medicine (patients 19 years and over), pediatrics (preschool and school children, 0–19 years), and gynecology (females 15 years and over). Every person has the right to freely choose one doctor in a certain field of work (general practice (GP), pediatrics and gynecology), according to the place of residence. Additional support for the work of chosen doctors is provided by other specialists employed in PHC (internal medicine, ophthalmology, psychiatry, radiology, laboratory diagnostics, etc.), but none of them fall into the category of a chosen physician. The stimulation for providing preventive health services among chosen physicians (GPs, pediatricians, and gynecologists) could be observed through capitation, a new model for payment introduced in PHC institutions in 2013. Capitation combines a fixed bigger part of the doctor’s salary and a much smaller variable part (cannot overcome 8.08% of the salary) that is performance-based and evaluated through four elements: the number of registered patients, efficiency, diagnostic–therapeutic procedures, and the quality of health care [9]. There are numerous preventive services under the capitation formula that include individual and group health promotion and education activities, immunization, the control of blood biomarkers, anthropometric measurements, risk assessment for NCDs, counseling, clinical examination and breast examination in women, and screening tests for the early detection of malignant diseases (for cervical, breast, and colorectal cancer) [9,13]. Health services in PHC are not paid except for a fixed co-payment for health services (50 RSD equivalent to ¢50) that is paid “out of pocket” by the insured if they are not exempt. Measures for the prevention and early detection of a disease are completely exempt from participation for all insured persons [14]. The content and scope of preventive services could indicate the quality of performance of PHC institutions and also give information about the activities of chosen physicians in the provision of health care services [14]. Data from 2017 showed that the lowest percentage of preventive check-ups in the total number of check-ups was provided by GPs (3.5%), followed by pediatricians (21.4%), while the highest percentage was found among gynecologists (42.2%) [15].

Studies worldwide have shown that the provision of preventive health services is associated with numerous sociodemographic (sex, type of settlement, age of the population, fertility rate, level of education, average salary, health care allocation, unemployment rate, etc.) and health system factors (representation of professional staff, their clinical practices, availability of PHC, and organization of the health service) [16,17,18]. Social determinants of health (SDH) represent the socio-economic situation in which people live and work, and they generate health inequalities between and within countries [2]. WHO guidelines are tailored at reducing the consequences of the unequal distribution of SDH that lead to unfair access to health care, pose a risk of disease, are complementary to the development of health systems, and even responsible to a great extent for health inequalities [17]. Therefore, actions should be directed outside the health sector towards strengthening the socio-economic status, which should not be a privilege or a limiting factor in the use of all health care services. Improving only one SDH can be a driver of the use of health services and a powerful way to improve health and better quality of health outcomes [18]. The results from the Republic of Serbia have shown that low educated males visited a GP significantly less often than their highly educated counterparts, while those who are older are more likely to have visited GP [19]. However, no research has been done to explore the factors influencing the use of preventive services in the Republic of Serbia; therefore, the aim of this study was to explore the availability and performance of health services in PHC (general practice, pediatrics, and gynecology) and to analyze the influence of sociodemographic and health determinants on the utilization of preventive health services in Republic of Serbia.

## 2. Materials and Methods

### 2.1. Study Design and Sample

This descriptive study used data from the National Health Insurance Fund and the Statistical Office of the Republic of Serbia for 2015. The units of observation were municipalities with their catchment areas. All 149 municipalities in the Republic of Serbia that have independent PHC units were included in the analysis. Following the Health Care Law [20] and the Decree on the Plan of the Network of Health Institutions [21], the founder of PHC institutions is the Republic of Serbia or autonomous province (for those on the territory of an autonomous province). Depending on the number of inhabitants of the municipality, the necessary health personnel is planned. 

The study was approved by the Ethics Review Board of the National Health Insurance Fund (Decision No. 450-1500/19, dated 7 March 2019).

### 2.2. Variables

The database of the National Health Insurance Fund was obtained upon request, formed on a quarterly level, and contains data of all PHC institutions regarding the availability and performance of chosen physicians (5024 in total for general practice, pediatrics, and gynecology) who are registered for providing medical services within the compulsory health insurance scheme. It provides data for the number of physicians included in performance-based payment (PBP), a number of registered users, and the number of provided health services (total and preventive) in PHC. Data for all four quarters of the year were merged according to the identification number of the physician and then summarized per municipalities. 

The database of the Statistical Office of the Republic of Serbia consists of data from municipalities and health surveys of the Republic of Serbia [22]. It was used for the calculation of socio-demographic (e.g., sex, population density, population aged 65+, urban population percentage, primary school completion rate, computer illiteracy - proportion of population without information and communication technologies (ICT) skills, unemployment rate, share of social services’ users in total population - users of accommodation services in foster families, care homes for elderly, users of social benefits like child allowance and financial social assistance, users of protection services for victims of domestic violence and children in conflict with the law, monthly net earnings per capita, health expenditure per capita, flats built per 1000 inhabitants, and life expectancy at birth for both sexes) and health indicators (e.g., fertility rate, perinatal mortality, under-5 mortality rate, cardiovascular diseases mortality rate, cancers mortality rate, respiratory diseases mortality rate, diabetes mortality rate, cervical cancer mortality rate, overall mortality rate, and NCD mortality rate). The following NCDs are available in the database: cardiovascular diseases, cancers, respiratory diseases, and diabetes. The database contains estimated population size by age, which was used to calculate target population sizes for general practice (adults aged 19 years and more), pediatrics (children aged 0–18 years), and gynecology (women aged 15 years and more).

Indicators of the availability and performance of chosen physicians in the area of preventive health services were calculated separately for general practice, pediatrics, and gynecology. The availability of chosen physicians was assessed as the number of chosen physicians in PHC per 10,000 people, population per physician in PHC, registered users per physician in PHC, and proportion of a target population registered with a chosen physician in PHC. The performance was assessed as the number of preventive services in PHC per 1000 people, number of health services per physician in PHC, number of preventive services per physician in PHC, and the proportion of preventive services within the total number of services delivered by a chosen physician. 

### 2.3. Statistical Analysis

Continuous variables are described with means and standard deviations or medians and interquartile ranges where appropriate. The relationship between the dependent and independent variables was analyzed by bivariate and multiple linear regression with backward elimination. Regression models were developed separately for domains of general practice, pediatrics, and gynecology. Multiple linear regression models included independent variables that were statistically significantly associated with a dependent variable in bivariate regression analysis. The results of linear regression analyses are presented with beta standardized coefficients and *p* values. The evaluation of multiple linear regression models is presented with coefficients of determination (r^2^). Statistical significance was tested at the level of 0.05. IBM Statistical Package for the Social Sciences (SPSS), version 21.0 for Windows was used for statistical analysis (IBM, Armonk, NY, USA).

## 3. Results

Detailed socio-economic and health characteristics of the population are given in Table 1.

The average population size in 149 municipalities in the Republic of Serbia for the year 2015 was 47,620 ± 5727.6, with a mean population density of 395.9 people per square kilometer (weighted mean was 92.5 people per square kilometer). The majority of the population completed primary school (completion rate of 94% or 97.6% weighted by population size), but computer illiteracy was found to be more than 50%, both weighted and unweighted. Monthly net earnings per capita were 345.6 USD, with a share of users of social services of 11.3% (9.3% weighted by population size). The unemployment rate was 11.5%. There was a wide variation in health expenditure per capita (213.7 ± 759.2 USD). Life expectancy at birth among women was 77.2 years, which was higher than the male population (72 years). The under-five mortality rate was 7.3 deaths per 1000 live births, while the overall mortality rate was 1685.8 deaths per 100,000 people. Cardiovascular diseases and cancer mortality rates were 933.3 and 305.3 per 100,000 people, respectively (Table 1).

A high variability between municipalities (SD over 50% of mean) was observed for population size (ranged from 4732 to 359,471 inhabitants); population density (ranged from 13 to 18,974 people per square kilometer); urban population (ranged from 0 to 100 percent); flats built per 1000 inhabitants (ranged from 0.1 to 8.8 flats per 1000 people); health expenditure per capita (ranged from 37.62 to 9308.66 USD); perinatal mortality rate (ranged from 0 to 67.6 deaths per 1000 births); under-five mortality rate (ranged from 0 to 44.1 deaths per 1000 live births); and respiratory diseases, diabetes, and cervical cancer mortality rates (ranged from 13.4 to 358.7, from 0 to 173.4, and from 0 to 98.3 deaths per 100,000 people, respectively).

Availability and performance indicators of chosen physicians in PHC are presented in Table 2.

The average population size per physician is larger than the average population registered per physician in PHC (1666.2 vs. 1155.6 for a GP, 1067.1 vs. 958.8 for a pediatrician, and 6481.4 vs. 2664.3 for a gynecologist). Findings showed that average proportions of the population registered with a physician were 69.4% for general practice, 89.9% for pediatrics, and 41.1% for gynecology. The number of health services per physician was 7234.1 for general practice, 6525.6 for pediatrics, and 5370.1 for gynecology. The proportion of preventive services within the total number of services provided in PHC was 8.8% for general practice, 18.1% for pediatrics, and 50.7% for gynecology. The number of preventive services provided per 1000 people was highest for pediatrics (1108.9), followed by gynecology (420.3) and general practice (381.9) (Table 2).

The results of bivariate and multiple linear regression analyses of preventive health services in PHC per 1000 people are shown in Table 3. Table 3 is divided into three sections presenting the results obtained for the domains of general practice, pediatrics, and gynecology.

In multiple regression model for the general practice domain, variables associated with the number of preventive health services provided per 1000 adults were health expenditure per capita (*p* = 0.006) and NCD mortality rate (*p* = 0.003), i.e., the higher health expenditure per capita and higher NCD mortality rate were, the more preventive health services were provided by a GP.

Regarding the pediatrics department, multiple regression analysis showed that municipalities where children had a higher completion rate of primary school (*p* = 0.024) had more preventive health services. Health expenditure (*p* = 0.017) and higher life expectancy at birth (*p* = 0.041) were also positively associated with the number of preventive health services.

In the gynecology domain, the only variable that was significantly associated with the number of preventive health services per 1000 women in multiple linear regression was the fertility rate (*p* = 0.033). The higher the fertility rate was, the more preventive health services were provided by a gynecologist.

## 4. Discussion

This is the first study to explore the availability and performance of primary health services (in general practice, pediatrics, and gynecology) and to analyze the influence of sociodemographic and health determinants on the utilization of preventive health services in the Republic of Serbia.

Our results showed that slightly more than two-thirds of the Republic of Serbian population is registered for a chosen GP. Though the level of health services provided by GPs is relatively high, only one in eleven services is preventive. Given the huge burden of the patients requiring curative services, GPs barely succeed in providing preventive services. The average of 10 min per patient limits the time that a GP can dedicate to their patients [23].

Nearly 90% of children in the Republic of Serbia are registered for a chosen pediatrician. This high number is the result of a well-coordinated connection between maternity yards and patronage services within PHC centers. The National Health Care Program for Women, Children and Adolescents [24] and the National Program for Supporting Breastfeeding, Family and Developmental Care of the Newborn [25] envisage a home visit by the patronage nurse for a new mother and new-born immediately after leaving the maternity yard. Within their scope of work, patronage nurses do home visits in the first days after birth, do counseling and demonstration about a baby and mother care, and provide information about the future baby’s check-ups including registration for chosen pediatricians [25]. Periodic check-ups are encouraged (at the age of 1, 3, 6, 9, and 12 months, among others), and pediatricians separate their working time into work with sick and healthy children [26].

The study showed a small number of the female population (41.1%) registered for a chosen gynecologist as a consequence of using the gynecology private practice services completely paid out of pocket. The main reasons are a shortage of gynecologists in primary health centers, long waiting times, and low levels of equipment [27]. 

According to the “Healthy People 2020” agenda [28], there are five key areas of SDH: economic stability, education, social and community context, health and health care, and neighborhood and built environment. As factors outside of the health system, that is, non-medical factors that have an impact on health outcomes, SDH account for between 30% and 55% of health outcomes. In addition, estimates showed that the contribution of sectors outside health to population health outcomes exceeds the contribution from the health sector [29]. The results of this study are being discussed in the light of a balance between improving care and investments in other SDH relevant to health.

Our study found that NCD mortality rate, health expenditure per capita, fertility rate, education (completion rate of primary school), and life expectancy at birth are significantly associated with preventive services utilization in PHC in the Republic of Serbia.

Regarding determinants associated with the use of preventive health service at the PHC level, a positive relationship between NCD and preventive service utilization was found in our study, which was in accordance with another study from Republic of Serbia [30] showing that there is a more than three times higher likelihood of having any GP visit among respondents with NCD multimorbidity compared with those with no disease. Van Oostrom et al. [31] noticed that patients diagnosed with multiple chronic diseases had more contacts with a GP on average than patients with one or without any chronic disease (19 vs. 11.2 and 5.3, respectively). A USA study [32] confirmed that people with colorectal cancer and comorbidities are more likely to have more contacts with their GP and therefore have greater use of preventive services. This is expected since preventive health check-ups are significantly important to those who suffer from chronic diseases. 

Our results indicated significant associations between health expenditures per capita and the use of preventive healthcare services in GP and pediatricians that follow the pattern of the higher expenditures are, the higher the likelihood of the use of preventive services is. Since income is the most important determinant of health care spending and there is no consensus on the other factors associated with the unexplained variation in health expenditure per capita, the use of preventive health services could be observed through the lens of income [33]. Our results were in line with previous research done in the USA and China confirming that the usage rate and frequency of preventive services are related to income, which further leads to higher health expenditure [34,35,36]. In the Republic of Serbia, those who belong to the most affluent socio-economic group were found to have visited a GP more often than disadvantaged ones. As Gwatkin et al. [37] noticed, as is typical for low income countries, the lack of health insurance and purchasing power among the worse-off mean that they consume health services—not only preventive but all health services—less often than the better-off. Studies from Europe have shown that people with higher incomes have higher odds of undertaking preventive examinations than poorer people [38,39]. In Germany, women with low socioeconomic status were less likely to use preventive services, including the identification of cardiovascular disease risk factors, examinations for diabetes mellitus type 2, and kidney disease [40]. The study by Garrido-Cumbrera et al. demonstrated that even in countries like Austria where health care is equally accessible to everyone regardless of socioeconomic status, inequalities in the utilization of primary preventive services exist [38]. People of lower income levels were found to be less likely to receive a preventive healthcare visit and fared worse in health outcomes [41,42].

In our study, educational achievement (completion of primary school) was found to be one of the influencing factors for preventive health service use at the department of pediatrics. This could be explained by the fact that more educated adolescents have better knowledge on the availability of services and benefits of preventive health care, and they have a higher receptivity towards new health-related information and better communication with their pediatricians. Education shapes future occupational opportunities and earning potential, and it provides knowledge and life skills that allow better educated persons to gain more access to information and resources to promote health [43], as well as a more proactive attitude in seeking health care. On the other hand, people with lower education may not recognize preventive care’s effect on health. Our findings were in line with the results of the study conducted in the USA confirming that the differences in educational attainment contributed to disparities in preventive service use [44]. A Hungarian study [12] showed that people with the highest education compared to those with completed primary education or less had a higher rate of using preventive health services such as screenings for hearing loss and visual acuity. An alternative interpretation might be that a higher use of preventive pediatric services may advance school completion.

The socio-economic differences in the utilization of health care could be explained by two major characteristics of health care systems, such as organization and modes of financing health care. It appears that the GPs position in the health system and the organization of primary care, as well as cost-sharing arrangements, are particularly important in this respect [45]. Given that timely and regularly implemented preventive examinations very often result in improved health outcomes. Preventive services during childhood are the cornerstone of preventive pediatric care in a way that promotes good health outcomes for children. Most pediatricians use these visits for monitoring early child development and focusing on disease screening. In our study, a higher life expectancy at birth was identified as one of the main factors for a higher number of preventive health services at pediatric departments. This was in line with the literature showing that preventive services focused on early disease detection can slow disease progression and improve outcomes [46]. Additionally, childhood immunization as a type of preventive service has the potential to save the most life-years [47]. The increased use of preventive services for children may prevent illness, reduce the long-term adverse health effects associated with some disorders, and improve health behaviors, with a net result of children who are more likely to become healthy, productive adults [48].

Our study indicated a strong association between fertility rate and the utilization of preventive health services at gynecology departments. The higher the fertility rate was, the more preventive health services were provided by a gynecologist. The fertility rate expressed as average children per woman usually reflects the number of pregnancies women had. According to WHO recommendations, healthy women with no underlying medical problems should have at least four preventive check-ups with their gynecologists during pregnancy [49]. Following the recommendations, it is expected that the higher number of pregnancies women had, the higher number of preventive visits she received.

The main strength of our study is that, to the best of our knowledge, this is the first research in the Republic of Serbia to document predictors of the utilization of preventive services in PHC. The study represents a secondary analysis of the nationally administrative data that gives particular weight to research. However, three limitations should be briefly stressed. The first refers to the impossibility to obtain information on some determinants like age, gender, type of settlement, and their relation to the utilization of preventive services since the data came from secondary databases. Secondly, the study is of limited explanatory power because it was impossible to examine the causal relationships between predictors and outcomes of interest. Thirdly, we did not consider the possible spatial autocorrelation of the phenomenon that adjacent geographical units (municipalities) are often more similar with each other than more distant ones, which may have affected statistical analysis by making observations dependent.

## 5. Conclusions

Our findings on the factors associated with the utilization of preventive health service at the primary level should serve as a starting point for where efforts should be made to achieve better health outcomes. The starting points in evaluating the quality of our health care can be observed from the angle of equality of the right to health and health care, as well as from the factors of access to health care. NCD mortality rate, health expenditure per capita, fertility rate, completion rate of primary school, and life expectancy at birth are the main predictors of preventive services utilization in PHC in the Republic of Serbia. The analysis of quality indicators of selected general practitioners, pediatricians, and gynecologists showed variations, while the work performance of physicians indicated an uneven level of the average number of health services provided at the primary level of health care in the Republic of Serbia. Decision and policymakers need to direct their activities and efforts to create and implement future strategies with a focus on increasing the number of preventive services at the PHC level. Therefore, there is a need to move from current care modalities based on treatment alone to ones with a focus on upstream interventions that would set the scene later for follow-up work that may help answers regarding the benefits derived from such an approach aimed at addressing NCDs.

## Figures and Tables

**Table 1:** General profile of the population and health status in 149 Republic of Serbian municipalities.

Characteristics	Mean ± SD ^a^	Weighted Mean ± SD ^b^
Population size	47,620 ± 5727.6	N/A
Percentage of males ^c^	49.5 ± 1.3	48.7 ± 1.3
Population density (number of population per km^2^)	395.9 ± 1844.5	92.5 ± 246.4
Population aged 65+	20.5 ± 4.2	18.7 ± 3.2
Urban population percentage ^c^	42.6 ± 27.8	59.1 ± 26.3
Primary school completion rate ^c^	94 ± 12.1	97.6 ± 11
Computer illiteracy (proportion of population without ICT skills) ^c^	58.5 ± 10.4	50.3 ± 11.1
Share of social services’ users in total population ^c^	11.3 ± 5.5	9.3 ± 4.2
Average earnings per capita (in USD)	345.6 ± 73.1	383.3 ± 83.8
Unemployment rate ^c^	11.5 ± 4.6	10.1 ± 3.8
Flats built per 1000 inhabitants	1 ± 1.2	1.7 ± 1.6
Health expenditure per capita (in USD)	213.7 ± 759.2	237.8 ± 671
Life expectancy at birth for both sexes	74.5 ± 1.3	75.1 ± 1.2
Life expectancy at birth for men	72 ± 1.6	72.6 ± 1.4
Life expectancy at birth for women	77.2 ± 1.2	77.7 ± 1.1
Fertility rate	1.4 ± 0.2	1.5 ± 0.2
Perinatal mortality (per 1000 births)	9.7 ± 9.1	8.8 ± 5.7
Under-5 mortality rate (per 1000 live births)	7.3 ± 7.2	6.3 ± 4.7
Cardiovascular diseases mortality rate (per 100,000)	933.3 ± 277.4	766.4 ± 223.7
Cancers mortality rate (per 100,000)	305.3 ± 62.4	301.3 ± 50.1
Respiratory diseases mortality rate (per 100,000)	94.7 ± 53.4	78.4 ± 38.3
Diabetes mortality rate (per 100,000)	51 ± 32.4	42.7 ± 27
Cervical cancer mortality rate (per 100,000)	12.3 ± 12.5	11.6 ± 8.3
Overall mortality rate (per 100,000)	1685.8 ± 377	1461.2 ± 301.4

^a^ Presented estimates indicate average levels across municipalities; ^b^ Cases were weighted by population size, except for population density which was weighted by the municipality area; ^c^ Estimates were calculated using the point values of the proportions as continuous variables; ICT: information and communication technologies.

**Table 2:** The availability and performance indicators of chosen physicians in primary health care in 149 Republic of Serbian municipalities.

Indicator		General Practice	Pediatrics	Gynecology
Number of physicians in PHC included in PBP	Median (IQR)	12 (9, 26)	5 (3, 10)	2 (1, 4)
Legislated minimum number of physicians in PHC ^a^	Median (IQR)	12.5 (7, 26.7)	3.9 (2, 8)	1.7 (0.9, 3.6)
Number of physicians in PHC per 10,000 people	Mean ± SD ^b^	6 ± 2.7	9.3 ± 2.9	1.5 ± 0.8
Population per physician in PHC	Mean ± SD ^c^	1666.2 ± 470.6	1067.1 ± 303	6481.4 ± 2603.3
Registered users per physician in PHC	Mean ± SD ^c^	1155.6 ± 251.8	958.8 ± 253.5	2664.3 ± 870.3
Proportion of population registered with a physician	Mean ± SD ^b^	69.4 ± 29.0	89.9 ± 20.7	41.1 ± 20.9
Number of health services per physician in PHC	Mean ± SD ^c^	7234.1 ± 1042.1	6525.6 ± 1145.9	5370.1 ± 1512.6
Number of preventive services per physician in PHC	Mean ± SD ^c^	636.3 ± 435.1	1182.7 ± 467.9	2724.1 ± 1066
Proportion of preventive services within total services provided in PHC ^d^	Mean ± SD ^e^	8.8 ± 5.7	18.1 ± 6.3	50.7 ± 12.9
Number of preventive services in PHC per 1000 people	Mean ± SD ^b^	381.9 ± 291.8	1108.9 ± 505.9	420.3 ± 230.5

^a^ Estimates were calculated using municipality population size and maximum number of registered users per physician given in national rulebook [14]; ^b^ Cases were weighted by target population (adults aged 19 years and more for general practice, children aged 0–18 years for pediatrics, and women aged 15 years and more for gynecology); ^c^ Cases were weighted by number of physicians; ^d^ Estimates were calculated using the point values of the proportions as continuous variables; ^e^ Cases were weighted by total number of services provided in PHC; PHC: primary health care; PBP: performance-based payment.

**Table 3:** Variables associated with the number of preventive health services in PHC per 1000 of the target population—bivariate and multiple linear regression analyses for domains of general practice, pediatrics, and gynecology.

Variables	General Practice				Pediatrics				Gynecology			
	Bivariate		Multiple		Bivariate		Multiple		Bivariate		Multiple	
	Beta	*p*	Beta	*p*	Beta	*p*	Beta	*p*	Beta	*p*	Beta	*p*
Population density	−0.054	0.513			0.228	0.006	^a^		0.237	0.006	^a^	
Population aged 65 and above	0.23	0.005	^a^		0.142	0.088			−0.112	0.198		
Fertility rate	−0.037	0.657			0.03	0.717			0.259	0.003	0.196	0.033
Urban population	0.01	0.909			0.256	0.002	^b^		0.191	0.028	^b^	
Completion rate of primary school	0.045	0.586			0.309	<0.001	0.2	0.024	0.217	0.012	^b^	
Computer illiteracy	0.018	0.827			−0.188	0.023	^b^		−0.264	0.002	^b^	
Share of social services’ users	−0.032	0.698			−0.194	0.019	^b^		−0.118	0.175		
Average earnings	0.006	0.945			0.089	0.285			0.271	0.002	0.163	0.081
Unemployment rate	0.04	0.632			−0.068	0.413			0.032	0.710		
Flats built per 1000 inhabitants	−0.075	0.397			0.043	0.633			0.168	0.067		
Health expenditure per capita	0.19	0.021	0.224	0.006	0.228	0.006	0.194	0.017	0.199	0.022	^b^	
Life expectancy at birth	0.057	0.488			0.256	0.002	0.177	0.041	0.178	0.039	0.149	0.082
Perinatal mortality	−0.006	0.938			−0.072	0.392			0.097	0.267		
Under-5 mortality rate	−0.14	0.088			−0.178	0.033	^b^		−0.058	0.506		
NCD mortality rate	0.212	0.009	0.243	0.003	NA				−0.247	0.004	^b^	
Model summary			r^2^ = 9.4%, *p* = 0.001				r^2^ = 15.1%, *p* < 0.001				r^2^ = 12.1%, *p* = 0.001	

^a^ Variable excluded from model due to multicollinearity; ^b^ Variable excluded from model using backward elimination. NCD: non-communicable disease.

## Data Availability

The data presented in this study are available on reasonable request to the corresponding author.
